# Bilateral Nasoalveolar Cyst Causing Nasal Obstruction

**DOI:** 10.1155/2016/4253090

**Published:** 2016-11-17

**Authors:** Uzeyir Yildizoglu, Fatih Arslan, Bahtiyar Polat, Abdullah Durmaz

**Affiliations:** ^1^Department of Otorhinolaryngology Head and Neck Surgery, Beytepe Military Hospital, Ankara, Turkey; ^2^Department of Otolaryngology, Head and Neck Surgery, Ankara Mevki Military Hospital, Ankara, Turkey; ^3^Department of Otorhinolaryngology Head and Neck Surgery, Gelibolu Military Hospital, Çanakkale, Turkey; ^4^Department of Otolaryngology, Head and Neck Surgery, Gulhane Military Medical Academy, Ankara, Turkey

## Abstract

Nasoalveolar cysts, which originate from epithelial remnants of nasolacrimal duct, are nonodontogenic soft tissue lesions of the upper jaw. These cysts are thought to be developmental and are presented with fullness in the upper lip and nose, swelling on the palate, and sometimes nasal obstruction. Because of cosmetic problems, they are often diagnosed at an early stage. These lesions are mostly revealed unilaterally but also can be seen on both sides. In this case report, a patient who complained of nasal obstruction and then diagnosed with bilateral nasoalveolar cysts and treated by sublabial excision is presented and clinical features and treatment approaches are discussed with the review of literature.

## 1. Introduction

Nasoalveolar cyst (NAC) is called nasolabial cyst or Klestadt's cyst. This developmental disorder which is defined as a nonodontogenic cyst of the upper jaw is seen four times more in women than men. It is mostly located unilaterally and left sided but rarely can be seen bilaterally. The patients are presented mainly in the fourth or fifth decade [1]. These lesions constitute about 0.7% of all jaw cysts. Typically, complaints of the patients are nasal obstruction and facial deformity. NACs, located in the hard palate and nasal vestibule, are presented with fullness of alar and nasolabial sulcus, submucosal mass with smooth surface on the hard palate, and bulging at the base of the nasal vestibule [2]. These cysts are usually painless but if infected, may cause pain and purulent discharge in nasal passages or in oral cavity. In this paper, due to rarity, a case of bilaterally located NAC is presented. The etiology, clinical features, and treatment approaches are discussed with the review of literature.

## 2. Case Presentation

**Figure 1:**
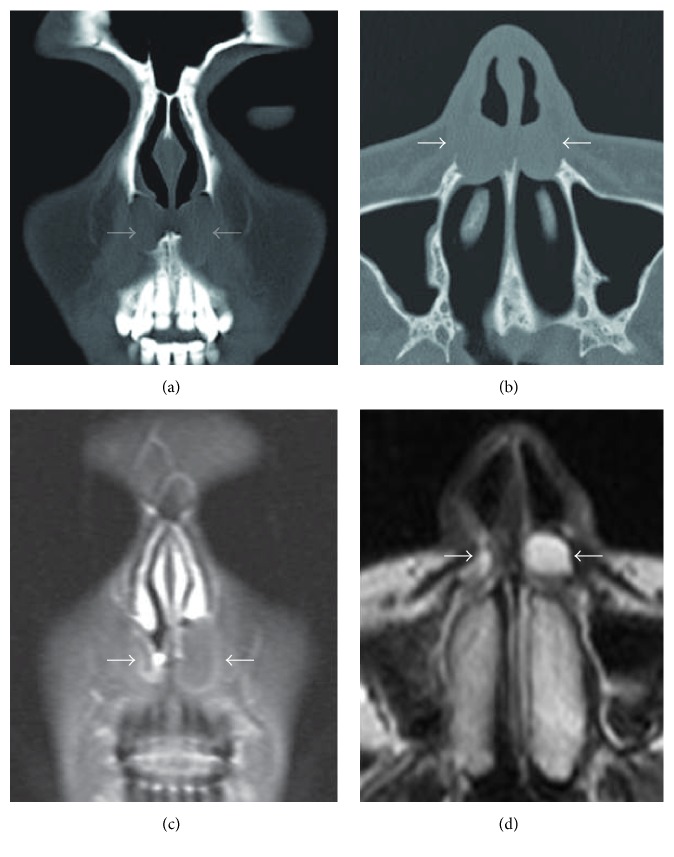
In the CT imaging of the maxillary bone, in both nasal vestibule floors, properly limited hypodense cystic lesions were observed, which were measured to be approximately 2 cm in the left and 1 cm in the right ((a) coronal view, (b) axial view). In the MR imaging performed after the antibiotherapy, at the same location, there were observed properly limited cystic lesions that did not show contrasting and looked hypointense at T1 display and hyperintense at T2 display ((c) coronal view, (d) axial view).

**Figure 2:**
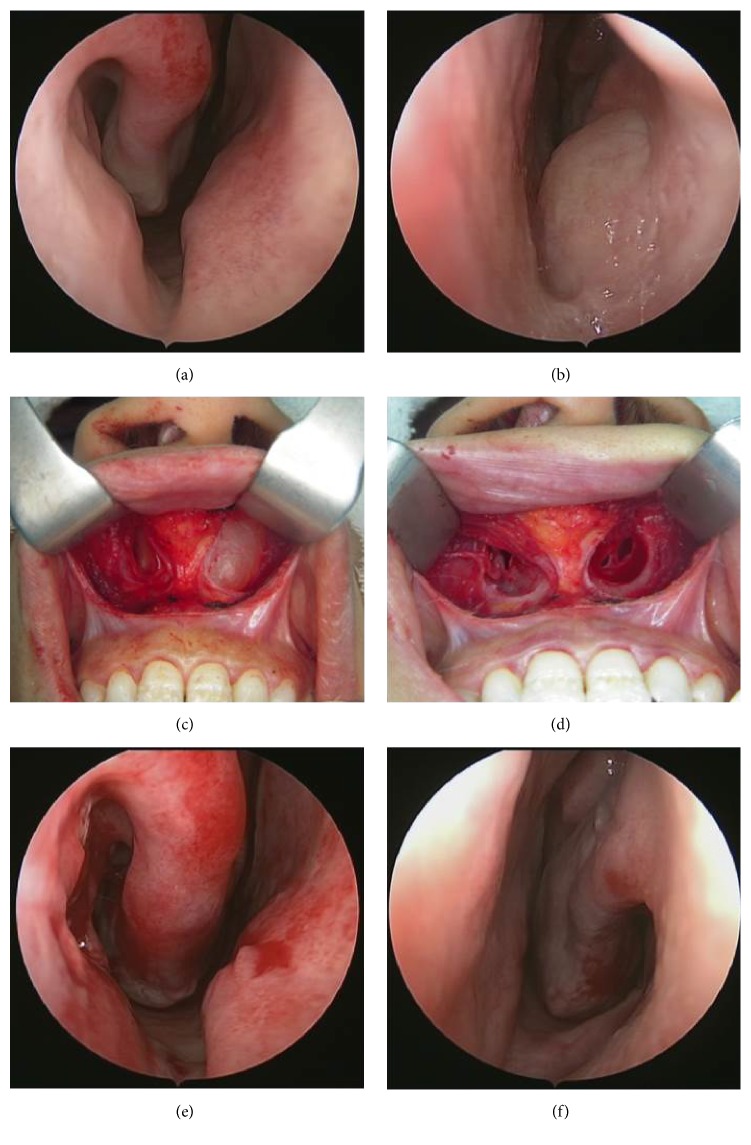
Intraoperative image of the cysts that were excised by sublabial approach. The cyst on the right was reduced in size because it ruptured due to an infection, but the cyst on the left kept narrowing the inferior meatus in the vestibule floor (a, b). In the intraoperative assessment fibrotic adhesions of the cyst on the right due to infection were seen, while the left cyst's borders were seen to be easily separated from the surrounding structures (c, d). After the cysts were totally excised nasal obstruction also passed (e, f).

**Figure 3:**
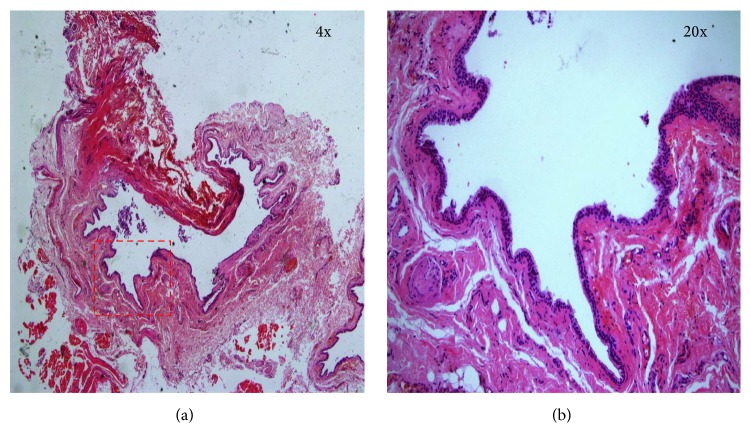
Light microscopy appearances of the cyst at 4x (a) and 20x (b) magnification. The cystic wall was lined with pseudostratified columnar epithelium.

A thirty-two-year-old female patient was admitted to our otolaryngology department with the complaints of having difficulty in breathing and rapidly growing painful swelling on the right side of the nose which has been existing for a few days and then caused purulent discharge by bursting. Anterior rhinoscopic examination revealed an infected cystic lesion at the base of the right nasal vestibule, towards the inferior turbinate by narrowing right nasal passage. A similar cystic lesion in the left nasal cavity was also observed, but it was painless and did not show signs of infection. Antibiotherapy (amoxicillin + clavulanate 1000 mg, 2 × 1, 7 d) and anti-inflammatory therapy were begun. In computed tomography (CT) images were obtained with the following parameters: field of view: 150 mm; section thickness: 1 mm; 200 mA 120 kV (Aquilion ONE 320, Toshiba Medical Systems Corporation, Japan). The CT scan showed well-bordered hypodense cystic lesions in both sides of the nasal vestibule floor which is about 2 cm in diameter on the left and about 1 cm in diameter on the right side (Figures 1(a) and 1(b)). The magnetic resonance (MR) imaging was made using Ingenia 3.0 T (Philips Healthcare, Netherlands) after the antibiotherapy. The MR imaging showed the well-circumscribed cystic lesions that did not show contrasting and looked hypointense at T1 display and hyperintense at T2 display at the same location (Figures 1(c) and 1(d)). The cysts were excised by sublabial approach (Figure 2). The right cyst has fibrotic adhesions due to previous infection, while the boundaries of the cyst in the left could easily be separated from the surrounding structures. Light microscopy of the cystic wall showed a connective tissue with few cells, lined with a flattened squamous epithelium. Microscopically, this cyst wall showed foci of chronic inflammatory cells and the wall was lined with two layers of squamous epithelium. The wall of the second cyst was lined with pseudostratified columnar epithelium (Figure 3). Both lesions were verified to be nasoalveolar cysts by histopathological examination. After the surgery no complications were observed and no recurrence was detected in the 6-month follow-up.

## 3. Discussion

Nasoalveolar cyst (NAC) is a congenital pathology and two theories have been developed about its etiology. The first theory is about cysts developing as nasolacrimal canal residues and the other is about the cysts being embryonic fissure cysts. When first described they were thought to develop from the retention of the mucosal glands [3]. Later, Kleinstadt suggested that these lesions developed from the tissue remaining in between during the merging of embryonic nasal mucosa, the maxillary process, and lateral-medial nasal process [4].

NAC usually is asymptomatic and is located next to the nasal wing. As the size of cyst increases it becomes symptomatic and usually causes swelling in the face and hard palate. The most common complaint is nasal congestion [2]. Fluid-containing serous-character NAC can become infected and drain into the nasal or oral cavity causing foul smell and pain in the mouth and nose. Although nasoalveolar cyst is a benign disease, it has been reported to be associated with malignant degeneration [3].

NAC is a soft tissue lesion which usually is placed inside the maxillary bone. Therefore CT imaging is the preferred method of viewing since it shows the location of the cyst and its relationship with the nasal and oral cavity and the status of the bone structure in a detailed way [5]. MR imaging has an advantage over CT imaging since it can show the content of the cyst and should be preferred as an additional survey in cases where malignancy is suspected. NAC does not cause destruction in the adjacent bone structures. However, the long term compression effect of the cyst can cause bone tissue erosion and even defects. In CT imaging, apart from these findings, sclerosing density increase around the lesion can be observed [3]. In our case the CT imaging examination showed a well-circumscribed cystic lesion. Due to compression in the surrounding bone structures, a smooth surface erosion was observed. In the differential diagnosis of the well-circumscribed cysts related to the anterior maxillary region, the nasal vestibule, and the hard palate, apart from the nasoalveolar cyst, the odontogenic cysts and the neoplastic lesions should also come to mind as well [6].

NAC is treated with surgical excision by sublabial approach [2, 3]. Other optional treatments include aspiration of the contents of the cyst, endoscopic cyst marsupialization by transnasal approach, and injection of sclerosing agents [2]. Lee et al. compared both groups of NAC patients, the ones who they treated by sublabial approach with the ones they treated by transnasal endoscopic marsupialization [7, 8]. According to these studies the transnasal endoscopic marsupialization was reported to be more advantageous when compared to sublabial excision since it shortened the surgery time, decreased the cost, and shortened the time of the postoperative pain. After the surgery no recurrence was reported in either groups. As a conclusion, transnasal endoscopic marsupialization was stated to be an advantageous and effective method in the treatment of NAC. Özer et al. reported a nasoalveolar cyst case that was treated by transnasal endoscopic approach and complete cyst excision was performed [9]. In our case, we aimed to remove the entire cyst wall instead of marsupialization and did excision by sublabial approach because of the localization, size, and infection history of the cysts. The part of the cystic wall which was adherent to the bone was delineated easily whereas the part of the cyst wall in the soft tissue of sublabial region was separated with difficulty especially in the infected cyst side. Thin cyst wall required slow dissection and sublabial approach permits good access to the pyriform aperture and wide exposure to the complete cyst.

Clinical and radiological findings are sufficient for the diagnosis of NAC but the definitive diagnosis is set by histopathological examination. Histopathological examination of NAC reveals cystic structure with fibrous capsule, which may contain goblet cells and is paved with pseudostratified columnar epithelium. As the size of the cyst grows, the intraluminal pressure increases and the columnar epithelium is replaced with stratified squamous epithelium. In our case, histopathological examination corrected the preoperative diagnosis compatible with NAC.

## 4. Conclusion

Bilateral NAC seems very rare and may cause nasal obstruction. When assessing breathing difficulties NAC should be kept in mind.
